# Exploring the Injury Severity Risk Factors in Fatal Crashes with Neural Network

**DOI:** 10.3390/ijerph17207466

**Published:** 2020-10-14

**Authors:** Arshad Jamal, Waleed Umer

**Affiliations:** 1 Department of Civil and Environmental Engineering, King Fahd University of Petroleum & Minerals, KFUPM BOX 5055, Dhahran 31261, Saudi Arabia; arshad.jamal@kfupm.edu.sa; 2 Department of Construction Engineering and Management, King Fahd University of Petroleum & Minerals, Dhahran 31261, Saudi Arabia

**Keywords:** road safety, crash injury severity prediction, machine learning, neural networks, sensitivity analysis, Saudi Arabia

## Abstract

A better understanding of circumstances contributing to the severity outcome of traffic crashes is an important goal of road safety studies. An in-depth crash injury severity analysis is vital for the proactive implementation of appropriate mitigation strategies. This study proposes an improved feed-forward neural network (FFNN) model for predicting injury severity associated with individual crashes using three years (2017–2019) of crash data collected along 15 rural highways in the Kingdom of Saudi Arabia (KSA). A total of 12,566 crashes were recorded during the study period with a binary injury severity outcome (fatal or non-fatal injury) for the variable to be predicted. FFNN architecture with back-propagation (BP) as a training algorithm, logistic as activation function, and six number of hidden neurons in the hidden layer yielded the best model performance. Results of model prediction for the test data were analyzed using different evaluation metrics such as overall accuracy, sensitivity, and specificity. Prediction results showed the adequacy and robust performance of the proposed method. A detailed sensitivity analysis of the optimized NN was also performed to show the impact and relative influence of different predictor variables on resulting crash injury severity. The sensitivity analysis results indicated that factors such as traffic volume, average travel speeds, weather conditions, on-site damage conditions, road and vehicle type, and involvement of pedestrians are the most sensitive variables. The methods applied in this study could be used in big data analysis of crash data, which can serve as a rapid-useful tool for policymakers to improve highway safety.

## 1. Introduction

Road safety has become a global public health threat in recent years. It is estimated that about 1.35 million people are killed, and over 50 million others are injured every year in traffic collisions worldwide [1]. As per the statistics from the World Health Organization (WHO) and World Bank, road traffic crashes (RTCs), on average, account for approximately 3% of the nation’s gross domestic product (GDP) worldwide, irrespective of their growth and rate of motorization [2]. A better understanding of factors contributing to traffic crashes is fundamental in improving crash prediction. However, RTCs are complex events involving many factors with multi-facet interactions, making it very challenging to comprehend them fully. Globally, various strategies have been successfully implemented to alleviate the burden of RTCs [3,4,5,6,7,8]. Intelligent traffic control and vehicle automation in urban areas are also aimed to ensure safe and sustainable traffic operation [9,10]. 

The Kingdom of Saudi Arabia (KSA) is located in Southwestern Asia. It is the largest country in the Arabian Peninsula, with an area of approximately 2.1 million square kilometers and a population of over 34 million. The entire area of the country is divided into 13 regions, having a mostly arid or semi-arid climate. KSA has a vast cultural diversity, with a significant proportion of the population belonging to expatriates from different parts of the world, particularly from South and East Asia and the neighboring Arab countries. The rate of urbanization is on the rise, with almost three-fifths of the population living in major cities that have integrated transportation services and access to basic services [11]. Due to rapid economic growth, particularly after the oil boom, the country has experienced an increased rate of motorization and congestion [12]. Vision 2030, announced in 2016, outlines 24 ambitious and specific goals for the political, economic, and societal domains. 

The enormous growth in motorization and road infrastructure has brought alarming road safety concerns in recent years in KSA. A recent study conducted by Turki et al. indicated that, on average, 19 persons are killed, and approximately four are injured in RTCs every day in the KSA [13]. Average crash to injury ratios of 8:6 and 8:4 are reported for the entire country and eastern region in the KSA, respectively, which are significantly high compared to the global ratio of 8:1 [14,15]. The economic losses due to RTCs in KSA are estimated to be around 4.3% of the national GDP [2]. In the literature, few studies have focused on identifying crash contributing factors to guide appropriate management strategies for mitigation [14,16,17,18]. Locally conducted studies suggest that factors such as driver distractions, over speeding, and aggressive driving, especially among young KSA adults, are the main factors that have contributed to an increase in crash occurrence and worsened injury severity [15,19,20,21]. In recent years, few road safety measures (such as the installation of the SAHER program, strict enforcement of traffic rules, the imposition of heavy fines on violators, etc.) have been initiated; however, the road safety situation has only marginally improved. 

As mentioned, the outcome of crash severity is significantly influenced by factors such as driver attributes, roadway characteristics, vehicle features, weather conditions, crash characteristics, and features of the built environment [22,23]. In the literature, various regression-based statistical models have been proposed to establish the relationship between crash injury severity and predictor variables. However, statistical models are built on several underlying observations and pre-defined associations among variables [24,25]. The weaker prediction performance of these models is another major concern that may be attributed to assumptions regarding linear link function and error distribution terms, which yield biased results if flouted. In recent years, machine learning-based models have emerged as a promising alternative to statistical methods in crash injury severity prediction. However, most of these studies have focused on overall prediction accuracy that does not improve the researcher’s understanding of the individual role of severity factors on injury severity outcome. To fill this research gap, this study proposes a neural network-based model of traffic crash incidents to predict the crash injury severity and evaluate the role of individual contributing factors on crash severity, in the KSA. Crash injury severity is under researched in the KSA. The rural highway crash data used in the analysis were obtained from the Ministry of Transport (MOT) at Riyadh. The findings of this study provide key insights for a better understanding of the factors contributing to fatal crashes along rural highways. The outcomes of this study are also expected to guide in identifying the critical factors heavily impacting the severity of crash accidents and proactively taking appropriate actions to mitigate them.

The rest of this paper is organized as follows. Section 2 provides a comprehensive literature review of different approaches related to crash injury severity modeling. Section 3 presents the data collection and descriptive statistics of the crash dataset. Section 4 highlights the methods utilized in this study. Section 5 provides the model prediction results and sensitivity analysis for severity risk factors using the proposed methods. Finally, Section 6 summarizes the main findings, study implications, and provide an outlook for future studies. 

## 2. Related Work

In the literature, crash injury severity analysis has been studied under two main headings (i.e., via statistical regression approaches (mostly used) and methods based on Machine Learning (ML)). The following passages provide a brief description of previous studies focusing on different statistical and ML approaches for crash injury severity prediction. 

### 2.1. Statistical Approaches in Crash Injury Severity Prediction 

Crash injury severity is usually represented by discrete categories such as fatal, incapacitating injury, capacitating injury, possible injuries, and property damage only (PDO). Due to the discrete nature of injury severity classes, it is often analyzed by discrete outcome statistical models such as binary, multinomial, probit, and logit models [26,27,28,29]. It is widely agreed that crash data may exhibit unobserved heterogeneity, which may be tackled by adopting other advanced statistical models such as ordered logit models [26,30,31,32], bivariate/multivariate models [33,34,35,36], random parameter model, [37,38], nested logit model [39,40], and Bayesian hierarchical models [41,42]. 

For example, Chen et al. employed probit models to identify factors contributing to crashes involving trucks [43]. The researchers found that factors such as driver’s gender, age, time of the crash, wet pavement surfaces, and adverse weather conditions were associated with higher crash severity. Hu et al. proposed a logit model to examine factors affecting crash injury severity at railroad junctions [22]. The study results showed that variables including the number of daily trips, presence of obstacle detection devices, and markings at approach segments significantly affect crash severity. Fan et al. compared the performance of ordered logit models and multinomial logit models for injury severity predictions of crashes at highway-rail crossings [44]. In his study, Mohamed Abdel-Aty analyzed the driver injury severity with ordered probit models and multinomial logit models [45]. The results showed that several factors contributing significantly to crash severity outcomes were common in both models like driver’s gender, age, seat belt use, speed ratio, point of impact, vehicle type, dark lighting conditions, presence of curves, etc. Comparing the modeling techniques, the ordered probit approach was more promising than the multinomial logit modeling method. 

Tulu et al. adopted a random-parameters logistic regression model for injury severity prediction of traffic crashes in Ethiopia [46]. Factors contributing to severe and fatal crashes included over speeding, night-time driving, collision with a heavy vehicle, and drivers with less educational background. Similarly, random parameter models were also investigated for crash severity modeling in other recent studies [38,47,48]. Kim et al. studied pedestrian injury severity in motor vehicle crashes with mixed logit models [49] and found that the odds of fatal crashes were increased significantly in the absence of street lights, collisions with trucks, speeding, drunk driving, and crashes occurring on freeways. Logistic regression is another widely used parametric approach in crash severity modeling. Jamal et al. used logistic regression to examine factors contributing to crash injury severity in the eastern province, the KSA [15]. The study reported that factors like driver’s distraction, over speeding, fatigue driving, sudden lane deviation, the involvement of pedestrians, and motorcyclists increased the injury severity. In their study, Meng et al. also utilized bi-level logistic regression to explore influential factors contributing to consecutive crash injury severity in Guizhou Province, China [50]. The study results showed that several predictor variables, including speed limit, traffic volumes, adverse weather conditions, and involvement of trucks, had a strong positive association with severe crashes. 

As mentioned, previous studies have utilized a wide range of statistical models for crash severity modeling. Though statistical models have a sound theoretical basis, they assume a pre-defined association between the variables, which, if flouted, yield erroneous model estimation. Alternatively, non-parametric methods of analysis have become popular in the road safety domain in recent years. 

### 2.2. Machine Learning Approaches in Crash Injury Severity Prediction 

To overcome the limitations of statistical models, ML approaches have been increasingly employed to model the potentially nonlinear relationships between crash severity outcomes and the contributing factors [51,52,53,54,55,56,57]. ML methods are more flexible with no or fewer model assumptions for input variables, and also have better fitting characteristics. Some of the commonly used ML approaches used in crash injury severity prediction include artificial neural networks (ANN) [58,59,60], random forest [54,61,62], support vector machines (SVM) [51,63,64], naïve Bayes [65,66,67], K-means clustering (KC) [68,69,70], and decision trees (DT) [71,72,73]. 

Several studies have developed different artificial neural network (ANN)-based models for crash severity prediction. For example, Delen et al. applied ANN to predict crash severity using eight binary input variables through features-based sensitivity analysis [74]. Study results demonstrated the robust predictive performance of proposed MLP networks for injury severity classification. Sensitivity analysis results revealed that the driver’s age and gender, use of seat belt and alcohol, and vehicle characteristics were found to have more influence on crash severity outcomes. Al-Kheder et al. applied ANN to predict the injury severity of crashes based on 5973 traffic crash observations from Abu Dhabi collected over a span of six years [75]. To enhance the prediction accuracy of ANN classifiers, data were split into three clusters via k-means algorithms. The target severity column was categorized into four severity groups (i.e., death, severe, moderate, and minor severity). The ANN classifier, with an average prediction accuracy of 74.6%, outperformed the ordered probit model with the corresponding value at around 59.5%. 

Random Forests (RF), Support Vector Machine (SVM), and Decision Trees (DT) are a few widely used ML techniques in crash severity analysis. Taamneh and Taamneh employed RF for predicting the injury severity of crashes based on six years of crash data from Abu Dhabi [76]. The data imbalance issue was tackled using SMOTE (Synthetic Minority Over-sampling Technique). For underrepresented classes (Severe Injuries and Death) in the actual dataset, the model performed poorly. Using the balanced dataset, the overall prediction accuracy of the model was around 78.5% indicating around 14% improvement. An ordered probit model was also used as a benchmark to validate the RF model. Mokhtarimousav et al. compared the SVM and random parameter mixed logit models for the severity prediction of work zone crashes [64]. Empirical findings revealed that prediction accuracy from SVM outperformed the mixed logit model. Analyzing the sensitivity of the parameters, variables such as the nature of termination areas in the work zones, type of activities, morning peaks, interstate highway types, and left-rear crashes all had a positive impact on crash severity. Wang et al. compared SVM with MLP and found that SVM achieved better prediction accuracy for crash injury severity [77]. 

Emhamed et al. implemented four ML algorithms, including RF, DT, Naive Bayes (NB), and logistic regression (LR), to predict crash severity prediction [78]. Findings indicated that all the algorithms yielded reasonable model performance. However, RF had the highest overall accuracy (75.5%) compared to logistic regression (74.5%), Adaboost (74.5%), and NB (73.1%). In another study, a researcher investigated the DT model for examining crash severity outcomes of motor vehicles using ten years from Missouri State [79]. The results suggested that factors such as alcohol use among drivers, over speeding, and failing to yield contribute significantly to fatal crashes. Delen et al., in their study, also compared four different ML methods (logistic regression, decision trees, NN, and SVM) to predict the injury severity of traffic crashes [80] and found that SVM achieved the highest prediction accuracy while logistic regression was the least accurate. The researchers noted that factors like collision type, non-compliance with seat belt, and drug involvement were the key contributors toward severe crashes. 

## 3. Data Description 

**Figure 1:**
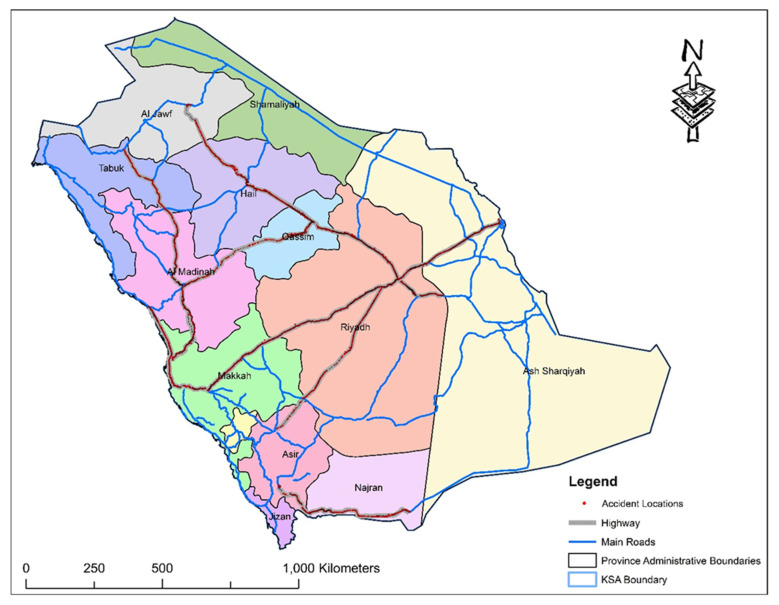
Locations of crashes in the study area.

Crash data used in this study were obtained from the traffic safety department at the Ministry of Transport (MOT), Riyadh, KSA, and covered three years (January 2017 to December 2019). The data was collected along 15 major rural highways (shown in Figure 1). A large proportion of selected highways runs through plain and desert terrain, having warm to high temperature during most part of the year. The database was compiled and extracted from the crash report file prepared by the on-site emergency response expert crew. The collected data were pre-processed and cleaned by removing the observations with outliers, duplicate records, and missing information. The final dataset contained a total of 12,566 valid crash observations resulting in 1320 fatalities and 7947 injuries. It included six main predictor variables categories (i.e., temporal, environmental, roadway, vehicle, traffic, and crash) with 59 sublevels (child features) for categorical variables and seven sublevels for continuous predictor variables. Table 1 summarizes the descriptive statistics of explanatory variables. Crash injury severity was classified into two levels (either fatal or non-fatal), which was the dependent variable. Table 2 provides the distribution by injury severity category across different years. Out of the total 12,566 crashes, 881 (7%) were classified as fatal crashes, and the remaining 11,685 were non-fatal crashes. Data about traffic volumes, road inventory, and others were also collected from the MOT.

## 4. Methods

Although a number of methods can be used to model crash severity for road data, this study chose artificial neural network (ANN)-based modeling because of its ability to learn patterns from the provided instances, with explicitly defining rules. ANNs try to mimic the way neurons in the human brain work to solve the problems and learn from the happenings around them [81]. There are numerous types of ANNs based on their architecture and internal working; however, this study entailed an improved feed-forward neural network (FFNN), which was trained using a back-propagation (BP) training algorithm. Generally, such networks comprise a single input layer, one or many hidden layers, and an output layer with varying number of neurons. For each example from the dataset, the input layer comprises of the input variables provided to the ANN. Before feeding the ANN, data for each input variable is normalized so that the absolute values of the different variables do not affect the performance of the ANN. Afterward, for a given ANN architecture, weights that define the relation between neurons of various layers are randomly assigned. Then, in each iteration, these weights are adjusted to minimize the error between the predicted output and the ground truth available from the training dataset. The adjustment of the weights is continued until a reduction in error is evident. Neural Designer (Artificial Intelligence Techniques Ltd., Salamanca, Spain) was used in this study for ANN implementation.

**Figure 2:**
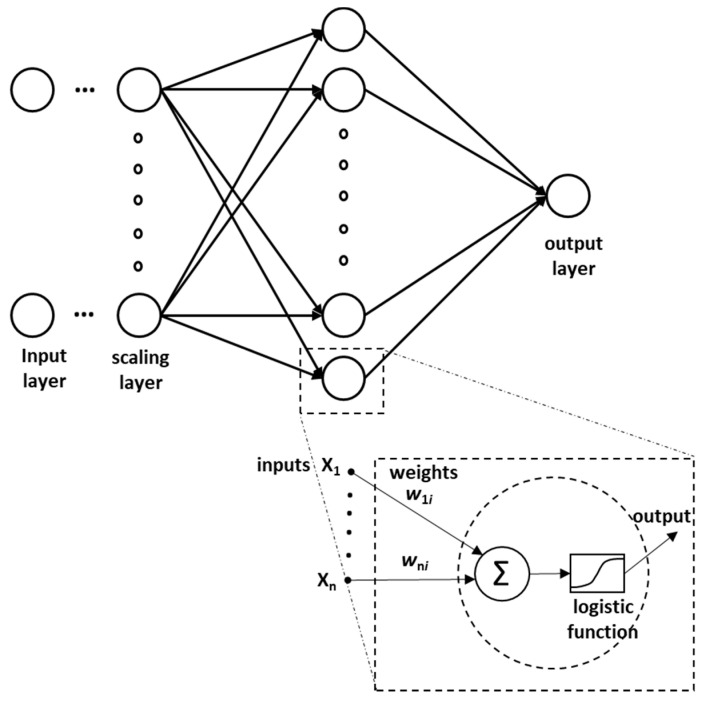
Artificial neural network (ANN) architecture adopted in this study.

While looking for an optimum architecture for the current problem, considering the data available and utilizing prior experience, the study limited the number of hidden layers to two with varying numbers of neurons. Then, the architecture was finalized based on the performance metrics during hit and trial runs. Accordingly, finally, the selected ANN comprises a single hidden layer with six neurons, as shown in Figure 2. Furthermore, since the purpose of the model was to predict/model crash severity, the activation function used in this study was the logistic function.

Since the performance of an ANN is heavily dependent upon the features being used, feature selection was conducted using correlation analysis between the available variables dataset and target variable (i.e., crash severity). Only features with a logistic correlation value greater than or equal to 0.04 were selected as the input features to the model. This resulted in a total of nine features to be used as input features (Accident type (*r* = −0.13), Weather Status (*r* = −0.12), ADDT (*r* = −0.08), Vehicle Type (*r* = 0.08), Number of Lanes (*r* = −0.05), Road Type (*r* = −0.05), Damage at Site (*r* = 0.04), Average Speed (*r* = 0.04), and Number of Vehicles Involved (*r* = 0.04)). These features were scaled to ensure the standardization of independent features so that all features are given equal importance initially. Training data for ANN comprised of 80% of the total dataset, whereas validation and testing datasets were both 10% of the total data. An important point to note was a skewed dataset (i.e., only 7% of the data represented fatal accidents). To handle this skewness, a weighted squared error was employed to train the ANN. Specifically, the error resulting from the wrong prediction of the fatal crash was penalized six times more than a non-fatal crash. The Quasi-Newton method was used to minimize the weighted squared error and optimize the ANN. Although it is based on Newton’s method, calculation of the Hessian matrix (second derivatives) is not required in this method, which is otherwise computationally expensive. Instead, the Quasi-Newton method approximately calculates the inverse of the Hessian matrix for each iteration using gradient information. In addition to mere prediction of crash severity, a mathematical model arising from ANN was further used to perform the sensitivity analysis. For this purpose, the base mathematical model was built with the following assumptions: (i) Road Type: Expressway; (ii) Weather Status: Shiny; (iii) Vehicle Type: Car; (iv) AADT: 3.55; (v) Average Speed: 2.76; (vi) Number of Lanes: 2.78; (vii) Accident Type: Crash; (viii) Number of Vehicles Involved: 1.44; and (ix) Damage at Site: No damage. Afterward, individual independent features were varied one-by-one to explore in-depth the impact of each feature variable on crash severity.

## 5. Results and Discussions

### 5.1. Model Performance Evaluation 

An ANN-based mathematical model for crash severity can be represented by Equation (1).

Crash_Severity = Logistic (6.83811+ (y_1*5.79538) + (y_2*-2.85844) + (y_3*-2.70326) + (y_4*-3.6051) + (y_5*-4.46448) + (y_6*-2.74106)) (1)

In the equation, Logistic refers to the application of a logistic function on the following mathematical expression. *y_1* til *y_6* represents the first and the second neuron of the hidden layer, which can be computed using the mathematical model given in Appendix A. Table 3 shows the confusion matrix and model predictive performance using different classification evaluation metrics for the test dataset. The confusion matrix shows the consistency between the actual and predicted observations for individual severity classes in the dataset. In the contingency table, the rows denote the predicted number of cases for each crash severity class, while the columns indicate the actual number of observations for a given severity group. The cells along the diagonal of the confusion matrix provide accurate severity predictions, while the off-diagonal values imitate misclassifications that result in underestimation or overestimation of a specific severity class. As shown in Table 3, a total of 43 observations of fatal injury class were correctly classified as fatal injury, and 33 cases in this severity category were misclassified as a non-fatal injury. Similarly, 731 observations of non-fatal severity class were correctly classified as non-fatal, whereas 192 observations of non-fatal observations were wrongly predicted under the fatal injury category. The overall prediction accuracy was around 77.5%, indicating an acceptable model performance. The sensitivity and specificity values obtained from the confusion matrix (given in Table 3) also showed that the proposed method is robust in predicting crash injury severity. 

### 5.2. Sensitivity Analysis for Variable Importance 

The following sections describe the results of the sensitivity analysis. As discussed before, a base model was used for this purpose, which was followed by changing the values of the variables of interest in the model one by one, to comprehend the changes in severity due to each variable.

#### 5.2.1. Sensitivity Analysis for Type of Highway

**Figure 3:**
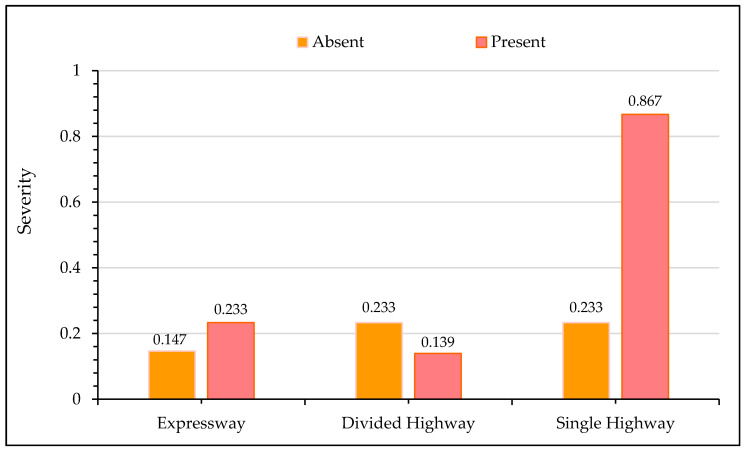
Effect of highway type on crash severity.

**Figure 4:**
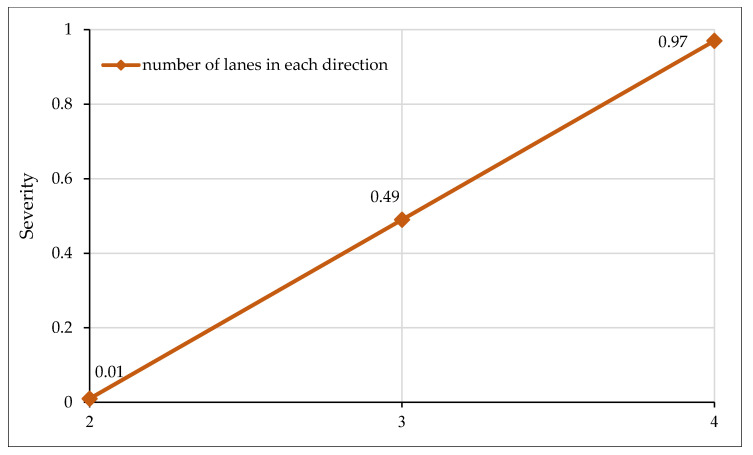
Effect of number of lanes in each direction on crash severity.

Figure 3 shows the impact of the highway type on crash severity with the *x*-axis representing the absence/presence of a specific highway category, and the *y*-axis displaying the severity outcome. Severity values close to “0” indicate that there are very low prospects of the crash being fatal, while a severity value approaching “1” represents a higher probability of a fatal crash occurrence. It may be noted from Figure 3 that the presence of an expressway increases the probability of a fatal crash by over 9%. For the current study, expressways were designated as the facilities with a minimum of three lanes in each direction and travel speed above 120 Km/h. Although freeways facilities have a forgiving design and are accompanied by protecting structures such as barriers, crash cushions, etc., the high travel speeds aggravate the impact during a crash event. The low traffic volume and forgiving road design along freeways sometimes also make the drivers more relaxed, which also intensifies the severity of crashes. These observations are intuitive and consistent with several previous studies [36,82,83]. On the other hand, the presence of a divided highway increases the chances of non-fatal crashes. A divided highway separates the traffic in the opposing direction by a median and has two lanes in each direction with a travel speed lower than expressways. Having relatively low speeds and high traffic volume could be the potential evidence for non-fatal crashes. Similarly, the majority of crashes reported on divided highways are rear end, which is also an indicator of non-fatal crashes [84,85]. Crashes occurring along a single highway have the highest probability of being fatal since there is no separating medium between the opposing traffic, and most of the crashes are head-on. It has been established that head-on crashes are associated with high fatality [86,87]. Figure 4 shows the relationships between the number of lanes in each direction versus observed crash severity. It is evident from the figure that severity increases as the number of lanes in each direction increases. With an increase in the number of lanes, drivers usually drive at high speed and are relatively relaxed, which could be one possible argument for increased crash severity. 

#### 5.2.2. Sensitivity Analysis for Weather Characteristics

**Figure 5:**
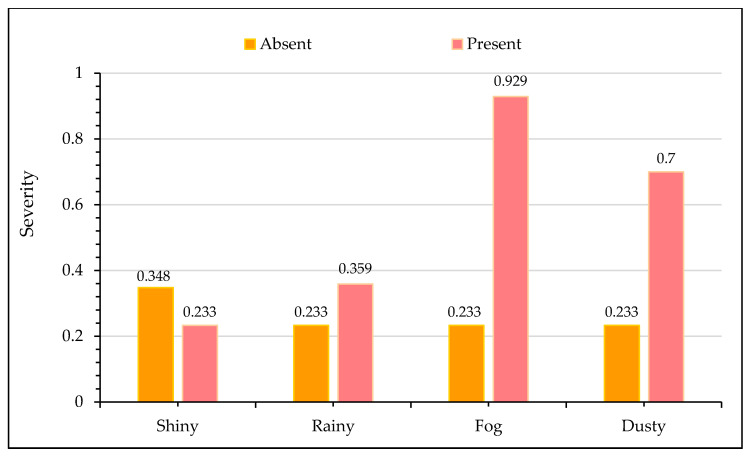
Effect of weather characteristics on crash severity.

Figure 5 portrays the effect of different weather conditions on crash severity output. It is worth noting that shiny weather conditions are associated with non-fatal crashes. During clear weather, drivers usually have a good sight of the roadway ahead during travel. Furthermore, they are more aware of adjacent vehicles and can detect the danger from a long distance, which gives them ample reaction time to control their vehicles. Thus, shiny weather reduces the prospects of severe crashes [88]. In contrast, the presence of adverse weather conditions such as rainy weather, fog, dusty conditions (mostly due to sand storms) all increases the likelihood of fatal crashes. High probability of fatal crashes during rainy weather conditions may be attributed to retarded visibility and loss of friction and pavement skid resistance [89,90]. Similarly, during foggy weather conditions, the driver’s sight is severely hampered, at times limiting the vision to only a few meters ahead. Drivers are unable to detect the nearby threat, and chances of fatalities are very high, even if vehicles are maintaining average traveling speeds since drivers do not have enough time to compensate. Sand storms are other prominent causes of fatal crashes throughout the KSA as they occur at different times of the year. During such weather conditions, the atmosphere is filled with dust and sand particles lasting from a few days to weeks, imparting poor visibility. These observations are in agreement with existing research. Several previous studies have reported that the presence of adverse weather conditions increases the injury severity of traffic crashes [89,91,92]. 

#### 5.2.3. Sensitivity Analysis for Vehicle Characteristics

**Figure 6:**
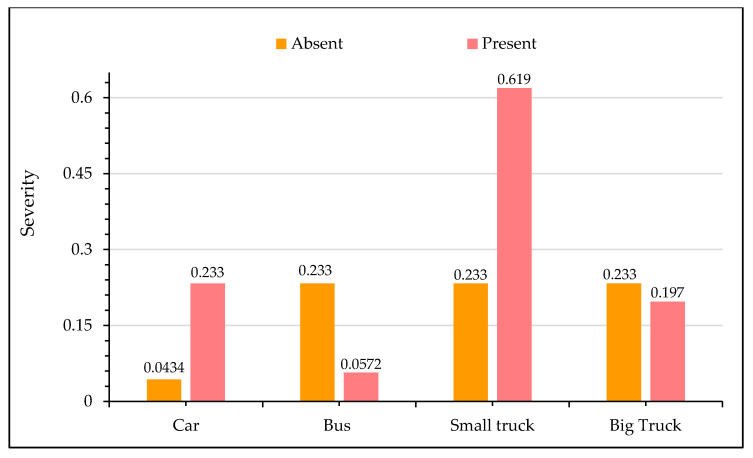
Effect of vehicle characteristics in crash severity.

**Figure 7:**
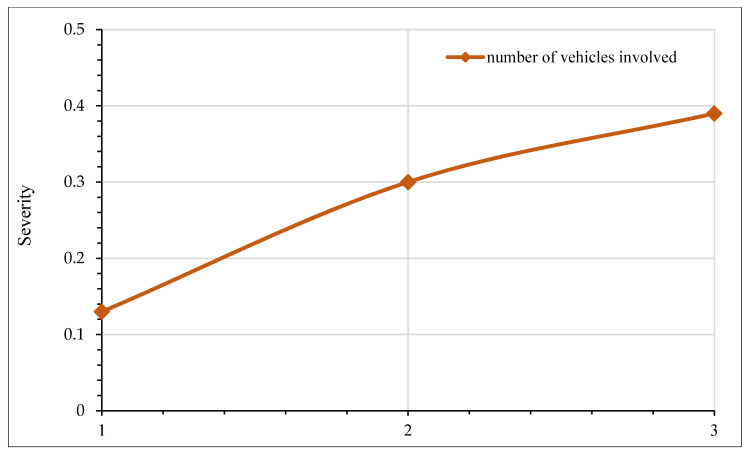
Effect of the number of vehicles involved in crash severity.

In Figure 6, sensitivity analysis for crash injury severity based on the type of vehicle involved is shown. It is evident from the figure that the presence of a car and a small truck is associated with an increased likelihood of fatal crashes. In particular, the probability of a fatal crash is increased by approximately 40% in collisions involving small trucks. This observation may be attributed to a severe impact due to the relatively high speeds of these vehicles. The literature suggests that high speed is an undeniable factor for greater kinetic energy release during motor vehicle collisions [93,94]. It is interesting to note that collisions involving buses and big trucks reduce the odds of fatal crashes. This perception is reasonable first because the average travel speed for buses and big trucks are relatively low, and second, these vehicles are structurally well-built and can absorb a significant amount of energy during the impact. Therefore, the chances of severe injuries are relatively less. These findings are consistent with the previous literature [95]. However, few studies have also reported that prospects for severe injuries are aggravated in crashes involving buses and trucks [49,96,97]. Figure 7 presents the relationship between crash injury severity and the total number of vehicles involved. It is clear that as the number of vehicles involved increases, there is a greater probability of a crash, resulting in a fatality. Earlier studies also suggest that multi-vehicle collisions are usually more prone to severe crashes [95,98,99]. 

#### 5.2.4. Sensitivity Analysis for Crash Characteristics

**Figure 8:**
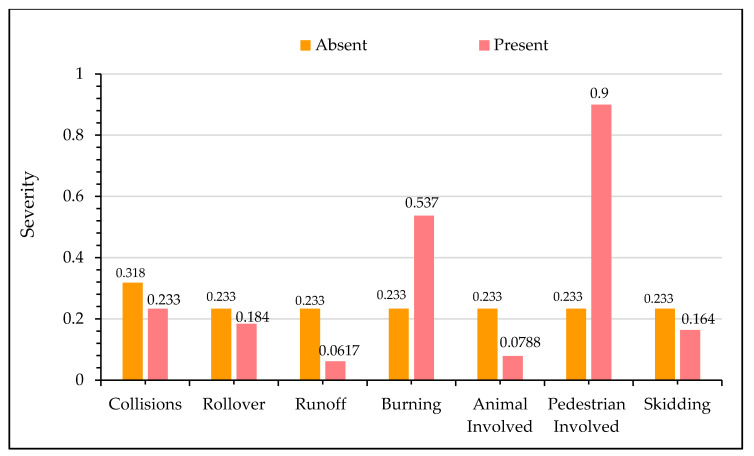
Effect of crash characteristics on crash severity.

Figure 8 shows the impact of crash characteristics on motor vehicle injury severity. As shown in the figure, crash types such as collisions between motor vehicles, vehicle rollover, run-off the road, crashes involving animals, and those due to skidding are associated with less severe crashes. While hit pedestrian crashes and those with vehicles burnt during the crash are likely to increase the odds of fatal crashes. In particular, crashes involving pedestrians increases the probability of fatal crashes by around 70%. During the collisions due to vehicle impact, the engine sometimes may catch fire, which can spread quickly to the entire vehicle, preventing quick rescue operations from evacuating drivers and passengers, who succumb to death [100]. Pedestrians are classified among the vulnerable road users’ group, are directly exposed to impact, which increases the chances of fatalities [101,102]. The observation that collisions between motor vehicles lower the probability of severe injuries may seem contradictory in light of existing research; however, it is worth mentioning that the majority of crashes reported for the current study were rear-ended, which mainly occurred along divided highways and expressways. The literature suggests that rear-end usually crashes, resulting in non-fatal injury [103]. The low probability of skidding crashes may be attributed to high driver alertness and lower speeds during inclement weather. Similarly, increased likelihood of minor injuries during run-off crashes may be credited to plain and greater recovery area along most highways in the KSA. Finally, there were only a few animals crashes in the dataset; this could be one of the potential reasons for a crash being non-fatal. 

#### 5.2.5. Sensitivity Analysis for On-Site Damage Conditions

**Figure 9:**
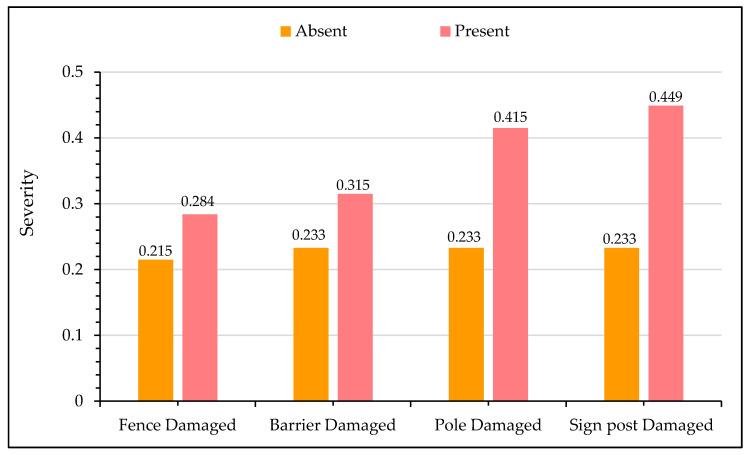
Effect of on-site damage condition on crash severity.

Figure 9 presents the sensitivity analysis for crash injury severity with on-site damaged conditions from crash locations. The presence of damage on the flexible barrier and fence is a hint of the slight increase in the likelihood of fatal crashes. The presence of a fence and side barrier dissipates and absorbs some of the energy of the errant vehicle and usually diverts it back to the highway. In comparison, the damage on signposts and poles are significant indicators of fatal crashes. Signposts and poles are usually located far from the main carriageway; thus, the presence of damage on them implies that either the vehicles were traveling at very high speeds or the driver fell asleep due to fatigue. Second, the structures of signposts and poles are very rigid and cannot absorb the kinetic energy upon impact, unlike flexible roadside barriers and fences. 

#### 5.2.6. Sensitivity Analysis for Traffic Characteristics

**Figure 10:**
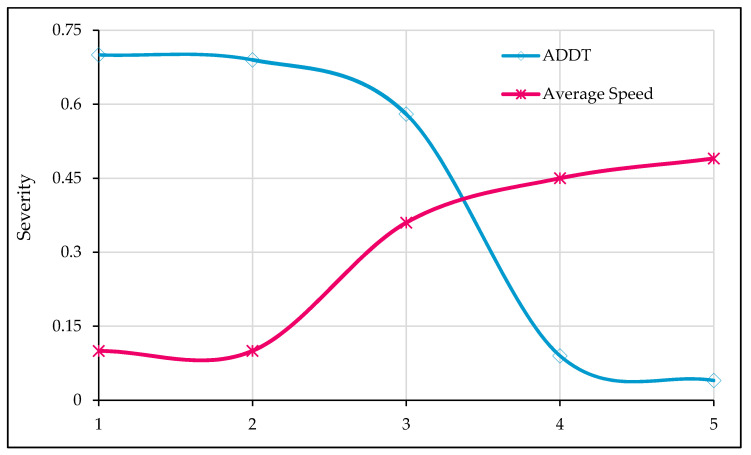
Effect of traffic characteristics on crash severity.

In Figure 10, the influence of traffic characteristics (AADT, and average stream speed) on crash injury severity is shown. The numeric values (1–5) for ADDT and average speeds shown on the *x*-axis represent the respective categories given in Table 1 (descriptive statistics). It may be noted from the figure that increasing AADT values are associated with non-fatal crashes. In contrast, high traveling speeds are indicators of fatal injury. Both these observations are intuitive and are in-line with the existing literature [86]. An increase in traffic reduces the drivers’ freedom to drive at free flow since their movement is restrained by other vehicles, who are bound to adjust their vehicle speeds. Furthermore, when ADDT is high, the drivers are more aware and alert to surrounding traffic. Many previous studies have established the association between high traveling speed and fatal crashes [93,104,105]. 

## 6. Conclusions 

Traffic crashes represent a threat to public health worldwide. Predicting crash injury severity is a promising research target in the highway safety domain. In recent years, ML-based methods have emerged as favorable alternatives to statistical methods due to their exceptional abilities to capture nonlinear relationships between variables, and fewer model assumptions, unlike the later ones. However, mere accurate prediction from ML does not advance the researcher’s understanding of the individual role of injury severity contributing factors, necessitating a thorough sensitivity analysis for predictor variables. In this study, an improved FFNN model was developed for injury severity prediction and variable sensitivity analysis using three years of crash data collected along rural highways in the KSA. A total of 12,566 crashes were reported that resulted in 1320 deaths and 7947 injuries. The processed data had six main categories of explanatory variables in 59 sub-levels for categorical variables. Injury severity, the target variable was classified into two severity groups (i.e., fatal and non-fatal injury). The overall prediction accuracy was around 77.5%, indicating an acceptable model performance given the extent and nature of the available input data. In addition, the results for classification metrics sensitivity and specificity also demonstrated the favorable predictive performance and efficacy of the proposed approach. Sensitivity analysis results via optimized NN architecture showed that variables such as traffic volume, average travel speeds, weather conditions, on-site damage conditions, road and vehicle type, and pedestrian involvement have a significant association with crash injury severity outcome. The findings of this study are expected to provide useful guidance to policymakers for adopting suitable countermeasures to enhance road safety. 

This study does have a few potential limitations that must be acknowledged. For example, this study was based on a limited three years of crash data with no detailed sociodemographic attributes of drivers that may have considerable influence on crash severity investigation. In the future, detailed datasets covering other road types, regions, and prolonged periods may be considered. Furthermore, this study considered only two severity groups; however, studies based on multiple injury severity classes may reveal interesting insights. Studies could also focus on the crash severity analysis of specific road user groups and collision types. Similarly, previous studies have found that crash data may involve many unobserved heterogeneity issues [106,107]. This problem may be tackled by dividing the data into several subgroups to uncover the relationship between crash injury severity and associated factors. Finally, severity prediction performance proposed methods could be compared with other advanced machine learning techniques (like ensemble learning, deep learning, Bayesian networks, etc.) and state-of-the-art statistical methods such as multivariate and random parameter models. 

## Figures and Tables

**Table 1:** Descriptive statistics of variables.

Variable Description	Variable Type	Categories	Frequency	Percent (%)
**Dependent Variable**				
Crash Injury Severity	Nominal	Fatal Injury	881	7
	Nominal	Non-Fatal Injury	11,685	93
**Explanatory Variables**				
**Temporal Features**				
Time of crash	Nominal	Peak	7087	56.40
	Nominal	Off-peak	5479	43.60
Day	Nominal	Weekday	9011	71.71
	Nominal	Weekend	3555	28.29
Season	Nominal	Winter	2754	21.92
	Nominal	Spring	1982	15.77
	Nominal	Summer	5313	42.28
	Nominal	Autumn	2517	20.03
**Environmental Features**				
Lighting Condition	Nominal	Day	7601	60.49
	Nominal	Night	4965	39.51
Weather	Nominal	Clear	11,003	87.56
	Nominal	Rain	519	4.13
	Nominal	Cloudy	234	1.86
	Nominal	Sand storm	364	2.90
	Nominal	others	446	3.55
**Roadway Features**				
Highway Type	Nominal	Divided Highway	2815	22.40
	Nominal	Expressway	9500	75.60
	Nominal	Single Highway	251	2.0
Alignment Type	Nominal	Tangent	8082	64.32
	Nominal	Horizontal curve	503	4.0
	Nominal	Vertical curve	205	1.63
	Nominal	Near intersection	132	1.05
	Nominal	others	3644	29.0
Surface Conditions	Nominal	Good	7107	56.56
	Nominal	Cracks	1257	10.0
	Nominal	Debris	454	3.61
	Nominal	wet	266	2.12
	Nominal	others	3481	27.70
Damage at Site	Nominal	Fence damaged	2615	20.81
	Nominal	Barrier damaged	1272	10.12
	Nominal	Pole damaged	498	3.96
	Nominal	Signpost damaged	307	2.44
	Nominal	others	7875	62.67
Shoulder width (m)	Numeric	Between 2.5–3.0	5312	42.27
	Numeric	Between 3.0–3.5	3402	27.07
	Numeric	Between 3.5–4.0	3853	30.66
Carriageway width (m)	Numeric	<7.5	1974	15.71
	Numeric	between 7.5–11	5319	42.33
	Numeric	>11	5274	41.97
Median width (m)	Numeric	<5	1177	9.37
	Numeric	Between 5–10	1061	8.44
	Numeric	Between10–15	1759	14.0
	Numeric	>15	8569	68.19
Road Markings	Nominal	Present	12,264	97.60
	Nominal	Absent	302	2.40
Road Cat eyes	Nominal	Present	12,398	98.66
	Nominal	Absent	168	1.34
**Traffic Characteristics**				
AADT	Numeric	<2000 (1)	476	3.79
	Numeric	Between 2000–5000 (2)	1282	10.20
	Numeric	Between 5000–10000 (3)	2983	23.74
	Numeric	Between 10000–20000 (4)	7136	56.79
	Numeric	>20000 (5)	687	5.47
Trucks % in ADDT	Numeric	<2%	607	4.83
	Numeric	between 2–5%	993	7.90
	Numeric	between 5–10%	1073	8.54
	Numeric	between 10–20%	6559	52.20
	Numeric	between 20–30%	3335	26.54
Average Speed (kmph)	Numeric	<90 (1)	529	4.21
	Numeric	between 90–100 (2)	2971	23.64
	Numeric	between 100–110 (3)	7467	59.42
	Numeric	between 110–120 (4)	1125	8.95
	Numeric	>120 (5)	474	3.78
**Vehicle Features**				
Type of Vehicle at Fault	Nominal	Car	7516	59.81
	Nominal	Bus	940	7.49
	Nominal	Small truck	1250	9.95
	Nominal	Big truck	2104	16.74
	Nominal	others	756	6.02
No. of vehicles involved	Numeric	1	6612	52.62
	Numeric	2	5518	43.91
	Numeric	>2	436	3.47
**Crash Characteristics**				
Collision Type	Nominal	Automobile Collision	6512	51.82
	Nominal	Hit Animal	174	1.39
	Nominal	Hit Pedestrian/	58	0.46
	Nominal	Rollover	3158	25.13
	Nominal	Run-off the road	1400	11.14
	Nominal	Skidding	98	0.78
	Nominal	Vehicle Burnt	296	2.36
	Nominal	others	870	6.92
Contributing Circumstance	Nominal	Driver (distractions, fatigue driving, disregard to traffic rules, and TCD)	9245	73.57
	Nominal	Animal	202	1.61
	Nominal	Faulty vehicle component	2120	16.87
	Nominal	Poor roadway	162	1.29
	Nominal	others	837	6.66

**Table 2:** Frequency and percentage distribution by injury severity category.

Year	Crash Injury Severity	Frequency	Percent (%)
2017	Fatal Injury	256	6.63%
	Non-Fatal Injury	3603	93.37%
	Total	3859	100%
2018	Fatal Injury	336	6.81%
	Non-Fatal Injury	4597	93.19%
	Total	4933	100%
2019	Fatal Injury	289	7.66%
	Non-Fatal Injury	3485	92.34%
	Total	3774	100%

**Table 3:** Confusion matrix for model performance evaluation.

Actual Severity Class	Predicted Severity Class		Accuracy	Sensitivity	Specificity
	Fatal	Non-fatal			
Fatal	43 (4.3%)	33 (3.3%)	77.5%	56.6%	79.2%
Non-Fatal	192 (19.2%)	731 (73.2%)			

## Appendix A

*y_1 = Logistic (-0.995587 + (scaled_expressway*-0.354336) + (scaled_divided_highway*-0.29182) + (scaled_single_highway*0.43358) + (scaled_shiny*-1.08956) + (scaled_rainy*-0.511944) + (scaled_fog*1.1712) + (scaled_dusty*0.894094) + (scaled_car*-0.418529) + (scaled_bus*-0.286795) + (scaled_smalltruck*-0.312071) + (scaled_bigtruck*0.687496) + (scaled_AverageAnnulaDailyTraffic_ADDT_*-0.782044) + (scaled_Avg.Speed*0.556496) + (scaled_NumberofLanes*0.395474) + (scaled_crash*1.18782) + (scaled_roll_over*-0.554596) + (scaled_Run_off_road*-2.23453) + (scaled_burning*0.222759) + (scaled_animal*1.35505) + (scaled_skidding*0.148185) + (scaled_pedestrian*0.359489) + (scaled_no_of_vehicles_involved*0.217981) + (scaled_no_damage*-0.0865889) + (scaled_fence*0.222342) + (scaled_barrier*-0.511662) + (scaled_lighting_pole*-0.0275431) + (scaled_signboard*-0.758213));*
*y_2 = Logistic (1.76863+ (scaled_expressway*2.37842) + (scaled_divided_highway*-1.83973) + (scaled_single_highway*0.14903) + (scaled_shiny*1.62146) + (scaled_rainy*0.576438) + (scaled_fog*1.33062) + (scaled_dusty*-2.01074) + (scaled_car*0.0929988) + (scaled_bus*-3.53022) + (scaled_smalltruck*0.398554) + (scaled_bigtruck*3.05684) + (scaled_AverageAnnulaDailyTraffic_ADDT_*-1.88497) + (scaled_Avg.Speed*3.50026) + (scaled_NumberofLanes*2.12327) + (scaled_crash*-0.586664) + (scaled_roll_over*-0.467537) + (scaled_Run_off_road*2.40548) + (scaled_burning*1.1775) + (scaled_animal*-1.07728) + (scaled_skidding*-1.07976) + (scaled_pedestrian*-0.50781) + (scaled_no_of_vehicles_involved*0.549078) + (scaled_no_damage*0.732242) + (scaled_fence*-2.44596) + (scaled_barrier*1.87162) + (scaled_lighting_pole*-2.40242) + (scaled_signboard*2.01491));*
*y_3 = Logistic (-0.150089 + (scaled_expressway*-0.779422) + (scaled_divided_highway*0.961344) + (scaled_single_highway*-0.396142) + (scaled_shiny*-0.803468) + (scaled_rainy*0.811967) + (scaled_fog*0.0198535) + (scaled_dusty*1.61784) + (scaled_car*-5.79697) + (scaled_bus*0.207224) + (scaled_smalltruck*4.38719) + (scaled_bigtruck*4.0538) + (scaled_AverageAnnulaDailyTraffic_ADDT_*1.1388) + (scaled_Avg.Speed*2.86474) + (scaled_NumberofLanes*3.69685) + (scaled_crash*2.13024) + (scaled_roll_over*-1.62119) + (scaled_Run_off_road*-1.87732) + (scaled_burning*-1.57074) + (scaled_animal*2.69996) + (scaled_skidding*0.659162) + (scaled_pedestrian*1.14106) + (scaled_no_of_vehicles_involved*0.678435) + (scaled_no_damage*2.04318) + (scaled_fence*2.63091) + (scaled_barrier*-4.31338) + (scaled_lighting_pole*0.515242) + (scaled_signboard*-1.34195));*
*y_4 = Logistic (1.09247 + (scaled_expressway*-0.987669) + (scaled_divided_highway*1.32038) + (scaled_single_highway*1.36756) + (scaled_shiny*-0.342812) + (scaled_rainy*1.35944) + (scaled_fog*1.7014) + (scaled_dusty*2.42403) + (scaled_car*3.65992) + (scaled_bus*-1.98591) + (scaled_smalltruck*-3.07896) + (scaled_bigtruck*-0.44773) + (scaled_AverageAnnulaDailyTraffic_ADDT_*5.01135) + (scaled_Avg.Speed*-0.581382) + (scaled_NumberofLanes*-5.97399) + (scaled_crash*0.469865) + (scaled_roll_over*2.85539) + (scaled_Run_off_road*-3.96823) + (scaled_burning*-0.851219) + (scaled_animal*-0.0944983) + (scaled_skidding*-0.150613) + (scaled_pedestrian*-0.655078) + (scaled_no_of_vehicles_involved*-0.534065) + (scaled_no_damage*-0.874981) + (scaled_fence*2.07124) + (scaled_barrier*-1.10295) + (scaled_lighting_pole*2.01337) + (scaled_signboard*4.01189));*
*y_5 = Logistic (1.31223 + (scaled_expressway*-0.460841) + (scaled_divided_highway*1.1601) + (scaled_single_highway*0.368057) + (scaled_shiny*1.20106) + (scaled_rainy*-0.342102) + (scaled_fog*-0.368233) + (scaled_dusty*0.648587) + (scaled_car*-0.168535) + (scaled_bus*0.903567) + (scaled_smalltruck*-0.972495) + (scaled_bigtruck*0.743239) + (scaled_AverageAnnulaDailyTraffic_ADDT_*-0.602835) + (scaled_Avg.Speed*-0.433562) + (scaled_NumberofLanes*0.184835) + (scaled_crash*0.163512) + (scaled_roll_over*-1.55079) + (scaled_Run_off_road*1.83793) + (scaled_burning*0.811734) + (scaled_animal*0.33973) + (scaled_skidding*0.640188) + (scaled_pedestrian*-0.217664) + (scaled_no_of_vehicles_involved*0.0232666) + (scaled_no_damage*0.129678) + (scaled_fence*0.923295) + (scaled_barrier*0.235469) + (scaled_lighting_pole*1.06563) + (scaled_signboard*-0.993238));*
*y_6 = Logistic (-1.73732 + (scaled_expressway*1.20981) + (scaled_divided_highway*-2.89906) + (scaled_single_highway*1.50868) + (scaled_shiny*-1.07576) + (scaled_rainy*-2.4256) + (scaled_fog*1.32154) + (scaled_dusty*0.900551) + (scaled_car*-5.39265) + (scaled_bus*2.01437) + (scaled_smalltruck*2.85767) + (scaled_bigtruck*2.69811) + (scaled_AverageAnnulaDailyTraffic_ADDT_*1.04841) + (scaled_Avg.Speed*-5.20317) + (scaled_NumberofLanes*3.78315) + (scaled_crash*0.463222) + (scaled_roll_over*-1.87714) + (scaled_Run_off_road*-0.591788) + (scaled_burning*0.80389) + (scaled_animal*3.39016) + (scaled_skidding*2.39256) + (scaled_pedestrian*-0.466654) + (scaled_no_of_vehicles_involved*-3.11739) + (scaled_no_damage*-0.414976) + (scaled_fence*0.38915) + (scaled_barrier*-0.164475) + (scaled_lighting_pole*-1.18927) + (scaled_signboard*-1.48262)).*
